# Pathogenic mechanisms and etiologic aspects of *Mycobacterium avium* subspecies *paratuberculosis* as an infectious cause of cutaneous melanoma

**DOI:** 10.1002/mog2.72

**Published:** 2024-05-12

**Authors:** Ellen S. Pierce, Charulata Jindal, Yuk Ming Choi, Kaitlin Cassidy, Jimmy T. Efird

**Affiliations:** 1 Independent Physician Researcher, Spokane Valley, Washington, USA; 2 School of Medicine and Public Health, University of Sydney, Sydney, New South Wales, Australia; 3 Provider Services, Signify Health, Dallas, Texas, USA; 4 VA Boston Healthcare System, Cooperative Studies Program Coordinating Center, Boston, Massachusetts, USA; 5 Department of Radiation Oncology, School of Medicine, Case Western Reserve University, Cleveland, Ohio, USA

**Keywords:** animal bacteria, infectious cancers, melanomagenesis, nonsolar, paratuberculosis, skin cancer, zoonotic etiology

## Abstract

Infectious etiologies have previously been proposed as causes of both melanoma and non-melanoma skin cancer. This exploratory overview explains and presents the evidence for the hypothesis that a microorganism excreted in infected ruminant animal feces, *Mycobacterium avium* subspecies *paratuberculosis* (MAP), is the cause of some cases of cutaneous melanoma (CM). Occupational, residential, and recreational contact with MAP-contaminated feces, soil, sand, and natural bodies of water may confer a higher rate of CM. Included in our hypothesis are possible reasons for the differing rates and locations of CM in persons with white versus nonwhite skin, why CM develops underneath nails and in vulvar skin, why canine melanoma is an excellent model for human melanoma, and why the Bacille Calmette-Guérin (BCG) vaccine has demonstrated efficacy in the prevention and treatment of CM. The pathogenic mechanisms and etiologic aspects of MAP, as a transmittable agent underlying CM risk, are carefully deliberated in this paper. Imbalances in gut and skin bacteria, genetic risk factors, and vaccine prevention/therapy are also discussed, while acknowledging that the evidence for a causal association between MAP exposure and CM remains circumstantial.

## 1 | INTRODUCTION

**FIGURE 1:**
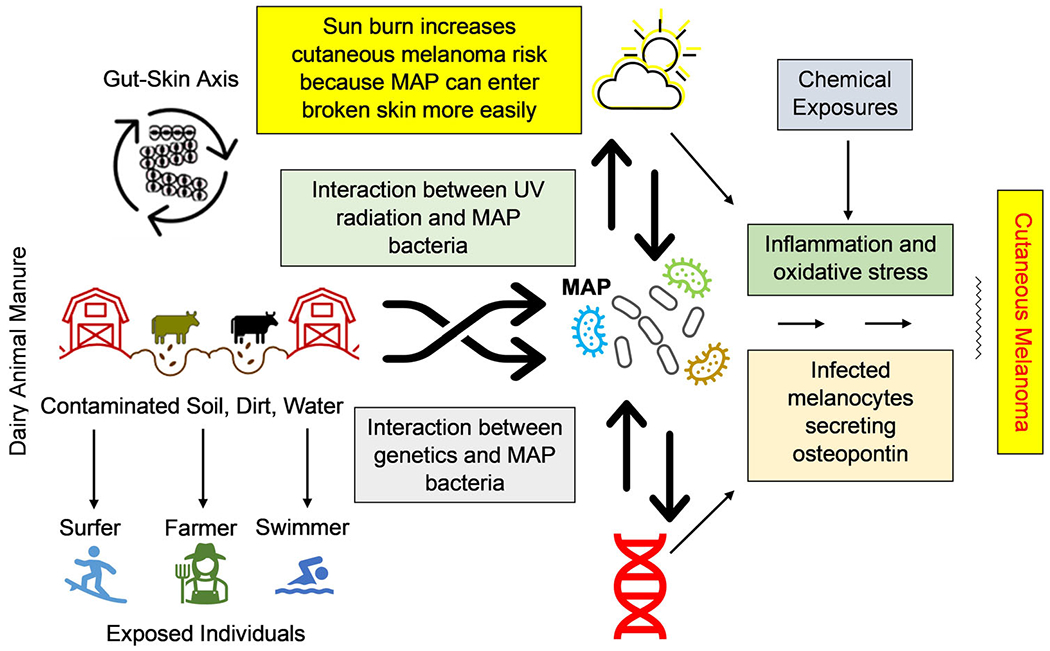
*Mycobacterium avium* subspecies *paratuberculosis* (MAP) exposure and related risk factors for cutaneous melanoma. Cutaneous melanoma is hypothesized to result from the interplay of MAP exposure, genetics, chemical exposures, and ultraviolet (UV) radiation. This combination leads to inflammation, oxidative stress, and infected melanocytes, setting the stage for cutaneous melanoma to develop.

Ultraviolet (UV) radiation is the most accepted cause of cutaneous melanoma (CM).^1–4^ However, up to 20% of CMs do not manifest UV-signature genetic mutations, suggesting that the interplay with *Mycobacterium avium* subspecies *paratuberculosis* (MAP) and other factors has an important role in this cancer (Figure 1).^5–7^

Known nonsolar risk factors for CM include chemicals such as pesticides^8^ and genetic conditions.^9^ CM often occurs in sites where the sun does not shine directly^10^ and moderate sun exposure is protective rather than detrimental.^11^ Indoor occupations generally confer a higher versus lower risk for CM.^12,13^ Another suspected nonsolar cause of human CM is the animal microorganism MAP. Besides MAP, exposure to other infectious agents, including human endogenous retroviruses^14,15^ and human papillomaviruses,^10,16,17^ may contribute to the development of CM.

Herein, we discuss the pathogenic characteristics and etiologic features of MAP exposure in the context of CM. Discourse is also focused on imbalances in gut and skin bacteria, genetic risk factors, and vaccine prevention/therapy, laying the groundwork for future hypotheses and research.

## 2 | PATHOGENESIS, ETIOLOGIC MECHANISMS OF MAP EXPOSURE, AND CM RISK

### 2.1 | Bacteria-related inflammation

The bacterial parasite MAP causes a chronic inflammatory enteropathy called Johne’s disease or paratuberculosis^18^ in large domestic ruminants such as dairy cattle and beef cattle,^19^ smaller domestic ruminants such as goats and sheep,^20^ wild ruminants and other nondomestic animals such as badgers, coyotes, crows, opossums, cats, rabbits, racoons,^21^ and primates.^22^ MAP also infects nonruminant animals including humans and is considered to be an “endemic trans-species pathogen”.^23^ This pathogen consistently has been associated with Crohn’s disease,^24–26^ to a lesser extent with other autoimmune diseases,^27–29^ and occasionally with human neurologic diseases and cancers.^30–34^ One may posit that chronic inflammation is a predecessor event for many cancers including melanoma.

### 2.2 | A role for MAP

A role for MAP in the development of CM previously has not been proposed, but is suggested by the following epidemiologic characteristics of CM. MAP is heavily excreted in an infected animal’s feces, and animal microorganisms excreted in an infected animal’s feces have been discussed as possible causes of farming and agriculture-associated cancers.^35,36^ With rare exceptions,^37^ a majority of studies describe an increased rate of CM in farmers and agricultural workers,^38–47^ although uncertainty exists regarding the strength of the associations. Studies have discussed that there is an excess of CM specifically in the faces, necks, and scalps of farmers^48,49^; three locations where MAP contaminated feces could easily reach otherwise clothed farmers. Solar radiation also may be an explanatory factor for these locations.^50^

A few studies show an increased rate of CM especially in cattle farmers. One study showed female dairy workers had the fifth highest occupational rate of CM.^51^ A majority of CM patients in two African studies were farmers or cattle herders.^52,53^ However, this could merely reflect the main occupation of the underlying population.

Veterinarians have an increased risk of CM^54–57^ and animal microorganisms have been proposed as possible causes for cancers identified in this occupational group.^35,46,55,58^ Two studies differentiated between “large animal” and “small animal” veterinarians, showing an increased risk of CM in the former.^55,59^ One study showed a high standardized mortality ratio for CM in large animal veterinarians, with cases occurring before the age of 30.^60^ Dairy and beef cattle are the primary animals that may be responsible for putting these types of veterinarians at an increased risk for CM.

**FIGURE 2:**
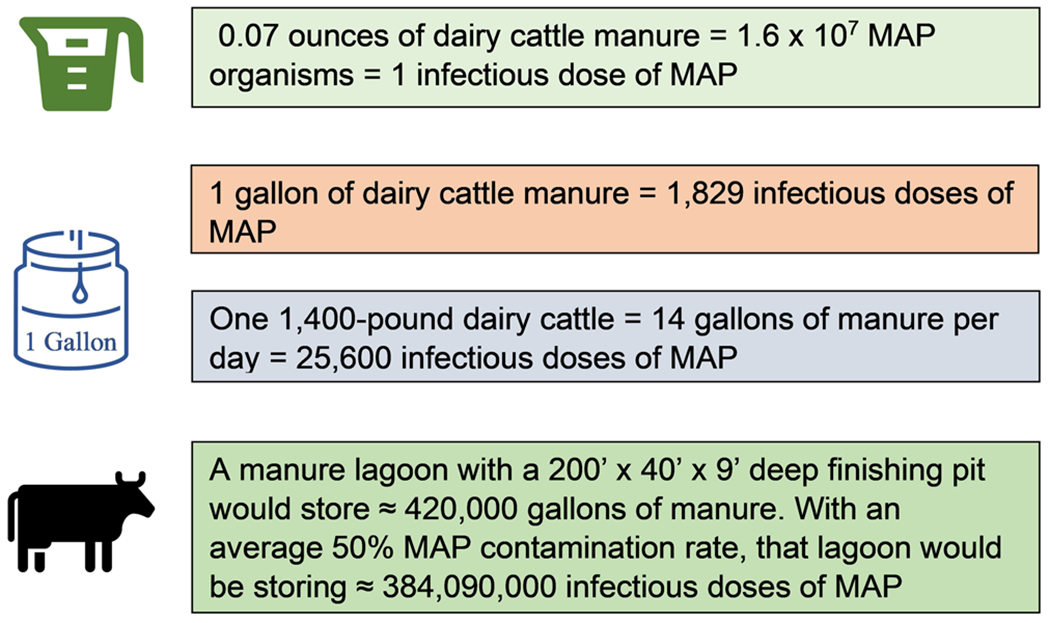
Concentration of *Mycobacterium avium* subspecies *paratuberculosis* (MAP) associated with cattle manure. Emphasis is placed on the relationship between the amount of manure and doses of MAP. With more manure it is expected that increasing doses of MAP are present.

Increased rates of CM in specific countries and regions within countries can be correlated with the presence of four main domestic ruminants: dairy and beef cattle, goats, and sheep. These animals often are located near bodies of water where their manure runoff causes water contamination. Higher rates of CM also have been observed in specific areas of the United States associated with considerable numbers of dairy cattle. Utah, Minnesota, Vermont, Idaho, and Iowa have the highest incidence rates of CM in the United States.^61^ All five states have massive numbers of dairy animals concentrated in confined animal feeding operations (CAFOs) or factory farms.^62–66^ The number of dairy animals per farm ranges from 200 to over 15,000 animals. All dairy operations in the United States with over 200 animals have at least one animal heavily excreting MAP organisms in its feces.^67^ This translates to significant amounts of exposure to MAP bacteria among residents in the vicinity of these farms (Figure 2).^68,69^ In Idaho, there has been a threefold increase in the incidence of CM^70^ associated with the development of CAFOs.^71^ A similar increase in CM in southern Arizona^72^ correlates with the establishment of dairy CAFOs in that particular region of the state.^73–75^

Furthermore, spatial clustering of CM near concentrations of dairy cattle with high rates of MAP infections has been documented in British Columbia, Canada.^76,77^ There are spatial clusters of high rates of CM in Ecuador^78^ associated with proximity to dairy cattle with high prevalence rates of MAP infection.^79^ The higher rate of CM in northern versus southern Italy corresponds with the increased concentration of dairy cattle in several northern provinces.^80^ A high rate of CM in southern Brazil^81^ is associated with prevalent dairy cattle farming.^82^

In theory, the high rate of CM among Australians may be explained not only by exposure to UV radiation but also exposure to sand and water contaminated by the feces of beef and dairy cattle and sheep. An increased rate of CM in coastal areas of Australia compared with inland areas,^83^ specifically in Queensland,^84^ overlaps with the 28 million beef cattle,^85^ 1.6 million dairy cattle,^86^ and 104 million sheep^87^ in Australia. These are concentrated along the coastlines in Queensland and New South Wales near swimming and surfing areas.^88,89^ One study localized the highest rate of CM to a specific section of southeast Queensland, precisely where the highest concentration of beef and dairy cattle are located.^90^

MAP organisms have been documented to remain viable in soil and dirt for over 1 year.^91,92^ In countries where MAP infections of domestic livestock are longstanding, MAP is present in soil samples throughout the country.^93^ The presence of MAP in soil and dirt may explain the increased risk of CM in chimney sweeps, specifically in their upper limbs, suggesting “skin contact with a harmful agent”^94^ and may underlie the increased risk of CM in outdoor athletes and sportsmen.^95^ As well, soil,^96,97^ sand,^98,99^ and natural bodies of water^100^ contaminated with MAP through manure runoff may explain the higher rate of CM in people exposed intermittently or occasionally to sunlight.^84,101^

“Melanoma is not considered to be associated with chronic sun exposure, but rather with heavy blistering overdoses, and is more common among non-manual workers of higher social status.”^47^ It may not be sunlight they are being occasionally or intermittently exposed to, but rather coming into contact with MAP through the activities they engage in when they are occasionally outside, including lying on and playing in MAP contaminated sand and water. This may explain the increased incidence of CM associated with “holidays spent at the beach.”^95^ Nonetheless, comparative studies are needed of CM in areas without heavy concentrations of cows.

Painful or blistering sunburns^102–104^ may increase the risk of CM because sunburn damages the epidermal layer of the skin, allowing MAP contaminated soil, sand, or water direct access to the melanocytes located in the epidermal–dermal junction. The association of trauma with melanomas may also be related to a similar break in the epidermal layer of the skin.^105,106^

Owing to MAP’s hydrophobic cell wall, all the MAP organisms in a body of water are concentrated at the surface.^107,108^ Aerosolization by the natural movement of water or by splashing and playing in water further concentrates MAP organisms because air bubbles scavenge MAP organisms from the surface of the body of water and then propel them into the air.^109–111^ The concentration of MAP, first at the surface of natural bodies of water and then their further concentration by aerosolization, is consistent with the increased rate of CM associated with occupational or recreational activities at the surface of bodies of water, including swimming,^112,113^ surfing,^113,114^ boating,^101,115,116^ and fishing.^101^ A recent study showing a 96-fold increased rate of CM in surfers (vs. an 18-fold increased rate in swimmers)^113^ is consistent with the forceful injection of aerosolized, concentrated MAP organisms into the skin. Alternatively, surfers might be more exposed to the sunlight/reflective rays while standing on their surfboards (vs. swimmers being more underwater). Questionably, CM rates may not be lower among surfers in areas with fewer cattle.

Several hypotheses have been proposed for the increased rate of CM in swimmers, including chlorine disinfection byproducts^116,117^ and UV radiation’s effect on organic matter creating hydrogen peroxide, which then damages hair follicles.^118^ An alternative explanation is the presence of MAP in both natural bodies of water contaminated with manure runoff,^100^ as well as in chlorinated potable water.^119^ MAP is known to be completely impervious to chlorine disinfection^120^ and is present in almost 90% of tap water samples in the United States.^121^

The presence of MAP in chlorinated water may explain the existence of vulvar melanomas, which are a subset of CM.^122^ It is difficult to see how UV radiation penetrates to the vulvar tissues, but very easy to see how MAP-contaminated chlorinated water does. One example is a case where a 68-year old woman with a vulvar melanoma^123^ reports over 900 h of swimming in indoor chlorinated water swimming pools, the bulk of which occurred before the age of 16 (Laura S. Brown, Ph.D., email communication, January 16, 2023). A common location of vulvar melanomas in the clitoris^124^ may be explained by the ability of MAP-contaminated chlorinated water to access the space underneath the hood of the clitoris.

UVB (medium wavelength, 280–315 nm) radiation is completely unable to penetrate the nail plate and UVA (longest wavelength, 315–400 nm) radiation only minimally.^125^ However, the ability of MAP-contaminated soil, dirt, and MAP-contaminated water, either chlorinated or natural water, to get underneath the nails, may explain the occurrence of subungual melanomas.

Metalworking fluids that are contaminated with MAP^126,127^ may explain the increased incidence of CM in the necks of welders,^128^ as the neck is where the spray of metalworking fluid reaches the skin. The increased risk of CM in petroleum workers and printers^129^ may be explained by the large amounts of possibly MAP-contaminated water used for crude oil extraction^130,131^ and in printing processes.^132^ Again, details pertaining to the exact levels of contamination in these studies are equivocal.

**FIGURE 3:**
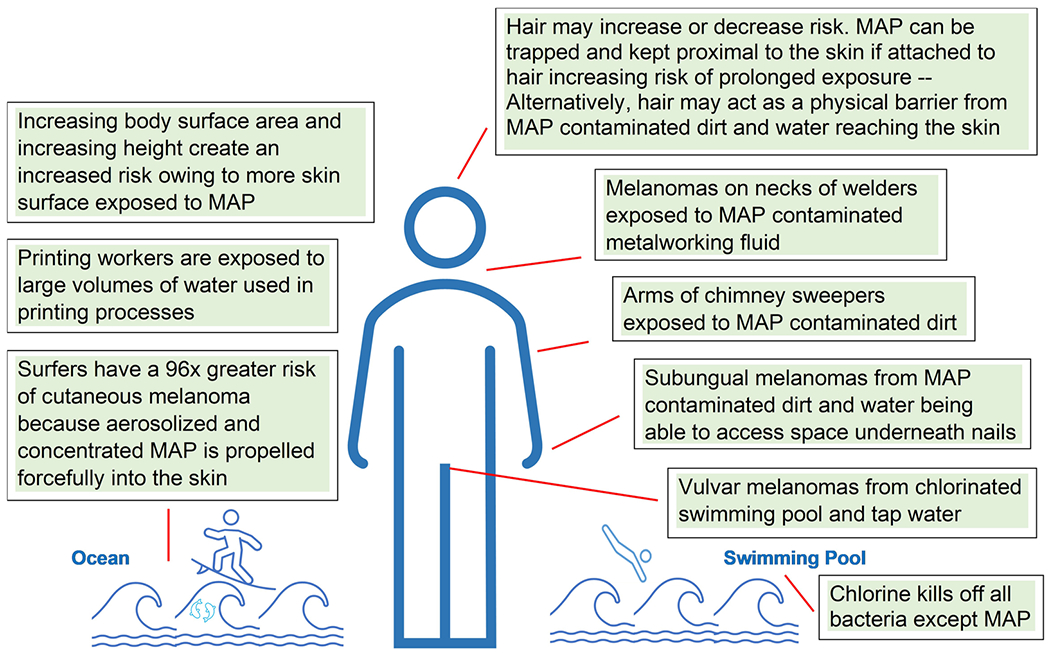
Potential alternative pathways for *Mycobacterium avium* subspecies *paratuberculosis* (MAP) exposure. Although MAP infection predominantly results from exposure to cattle manure where MAP is present, MAP infection can also occur from exposure to water or dirt contaminated by these bacteria. As illustrated above, both recreational and occupational activities can increase the risk of MAP exposure such as swimming, surfing, welding, cleaning chimney sweeps, and printing.

Hair cover may protect against invasive melanoma of the head, neck, and ear^133^ because MAP organisms may be clinging to the hair, rather than reaching the scalp, neck, or ear skin. Some studies have shown that obesity increases the risk of CM, specifically through increased body surface area rather than body mass index, perhaps because having more skin surface area increases the chances of MAP penetrating the skin.^134,135^ The increase in skin surface area with increasing height may also explain the putative association of CM risk with increasing childhood height.^136,137^ As well, the increase in surface area could be associated with both increased sun exposure and increased exposure to MAP. Potential alternative pathways of MAP exposure are summarized in Figure 3.

### 2.3 | Skin color

Individuals with white skin^138^ have a higher rate of CM, while individuals with nonwhite skin have a lower rate of CM. The location of CM in individuals with nonwhite skin differs from the location of CM in individuals with white skin. In insects, the production of melanin (melanization) is a response to bacterial penetration of the cuticle of the exoskeleton, and encapsulates the invading bacteria.^139^ Authors have suggested that human skin also acts as an antimicrobial barrier,^140,141^ and skin color has developed as a protective factor against microorganisms.^141,142^ “Animal husbandry practices” have been suggested as one pressure on the development of skin color.^141^ Skin color was a protective factor among Vietnam Veterans regarding bacterial and parasitic infections. Black Vietnam Veterans were less likely to develop such infections in comparison to white Vietnam Veterans.^141^

One study has shown the individual melanosomes that are the predominant type of melanosome in nonwhite skin are bacterial lysosomal degradative organelles, in contrast to the melanosome clusters in white skin that have no effect on bacteria.^143^ Darker skin tone corresponds to larger melanosomes and more effective antibacterial degradative organelle melanosomes.^144^ Individual melanosomes in hair follicles increase in size as a person ages.^145^ The possibility that individual melanosomes in skin may be smaller in children in contrast to adults may explain the greater risk of CM in children with nonwhite skin than adults with nonwhite skin.^146^ An alternative explanation is that children of any skin color are more likely to play in dirt than adults.

Individuals with nonwhite skin rarely develop CM. Only 1237 Asians with CM were identified in a United States database versus 409,000 non-Hispanic whites over the same time period.^147^ Another study identified over 48,000 non-Hispanic whites with CM versus 932 Hispanics whites, 394 Asian Americans and Pacific Islanders, 251 African Americans, and 52 Indigenous (First Nations) people.^138^ The only nonwhite skin that contains predominantly melanosome clusters are the palms of the hands and soles of the feet.^148^ This may explain the almost exclusive locations of these two CM sites in individuals with nonwhite skin.^149^ Both white and nonwhite individuals develop palmar and plantar melanomas,^150^ but nonwhite individuals rarely develop CM in any other location.

The increased rate of vulvar melanomas in white versus black women^151^ possibly suggests that the labial skin of black women may be another skin site that contains predominantly melanosome clusters rather than individual melanosomes.

### 2.4 | Additional considerations of MAP exposure

Farmers and swimmers are exposed to MAP in outdoor environments and are exposed to sunlight as well as MAP. UV radiation from sunlight may be immunosuppressive and indirectly increase the risk of infection with MAP.^152^ Conversely, UV radiation may directly cause melanoma independent of an MAP infection by means of systemic alteration in the immune system.^153,154^ Some alternative sources of UV radiation in addition to sunlight include tanning beds, artificial lighting (mercury vapor, halogen, fluorescent, and incandescent), and lasers.^155^

The exposure of farmers to pesticides has been implicated in their increased rate of cancer.^156^ We note that exposure to the herbicide glyphosate (Roundup^®^) may be linked to MAP-associated human disease^157^ owing to glyphosate’s ability to kill commensal but not pathogenic bacteria.^158–160^ However, we are not aware on any studies showing decreased CM risk with herbicides killing pathogenic bacteria.

In some cases, a direct, positive association may not exist for MAP exposure. For example, there is a reduced rather than increased incidence of CM in butchers presumably exposed to MAP contaminated meat and blood.^161^ Also, the association of CM with the leather industry is weak and has been linked to the chemicals used in leatherwork rather than exposure to animal microorganisms.^162,163^

### 2.5 | How MAP could cause CM

MAP’s mechanism of action may resemble cancers associated with other bacteria such as *Helicobacter pylori* (gastric cancer), *Salmonella enterica* (biliary cancer), and *Chlamydia trachomatis* (cervical cancer).^164–166^ Furthermore, it has been shown that *Mycobacterium tuberculosis* (MTB), with features similar to MAP, may promote oncogenesis through chronic inflammation resulting from increased levels of tumor necrosis factor-alpha (TNF-α).^167^ MAP, like MTB, is a persistent and asymptomatic infection.^168^ Approximately eight of nine individuals remain MAP positive 1 year after their first positive test.^168^

We propose an additional specific mechanism for MAP-associated CM. Recently, it has been observed that melanocytes are the precursor cells for CM and CM cells are malignant melanocytes.^169^ Conceivably, malignant melanoma cells are melanocytes that are chronically infected with MAP. Chronic MAP infection of cutaneous melanocytes may result in CM by the continuing secretion of osteopontin by MAP-infected melanocytes. Osteopontin is a glycoprotein secreted by cells infected with mycobacteria of different species. These species include MAP,^170–172^ *Mycobacterium avium intracellulare*,^173^ MTB,^174^ and the Bacille Calmette-Guérin (BCG) vaccine^173,175^ (an inactivated strain of *Mycobacterium bovis*).

Serum osteopontin levels are increased in patients with CM.^176^ Osteopontin enhances the host cell’s response to the infecting *Mycobacterium* by stimulating T helper cell type-1 secretion.^173^ Osteopontin is thought to stimulate epithelial to mesenchymal transition.^177,178^ Osteopontin also has antiapoptotic effects in melanocytes.^179^ It is possible that a melanocyte infected with MAP is chronically secreting osteopontin and that persistent osteopontin antiapoptotic stimulation results in the malignant transformation of infected melanocytes.^179^ Regional and distant metastases of CM may be equivalent to lymphatic and hematogenous dissemination respectively of MAP-infected melanocytes.^180^

MAP organisms typically reach melanocytes from the skin surface, rather than from an internal location. The increased rate of CM in patients with idiopathic inflammatory bowel disease, particularly Crohn’s disease,^181,182^ may be explained by skin contact with rather than ingestion of MAP-contaminated soil, unchlorinated water, or chlorinated drinking water. The ingestion of chlorinated tap water has been linked to a cluster of Crohn’s disease,^183^ but individuals with inflammatory bowel disease also are bathing and swimming in MAP-contaminated chlorinated water. On the other hand, the persistent ingestion and lingering of MAP may disrupt the gut microbiome leading to systemic inflammation and cancer. Additionally, Crohn’s disease conceivably could cause “holes” in the internal mucosal lining (similarly to what sunburn does on the surface of skin) that allow MAP from tap water to enter the system in a way that would not be possible with intact mucosa.

### 2.6 | Imbalances in gut and skin bacteria

**FIGURE 4:**
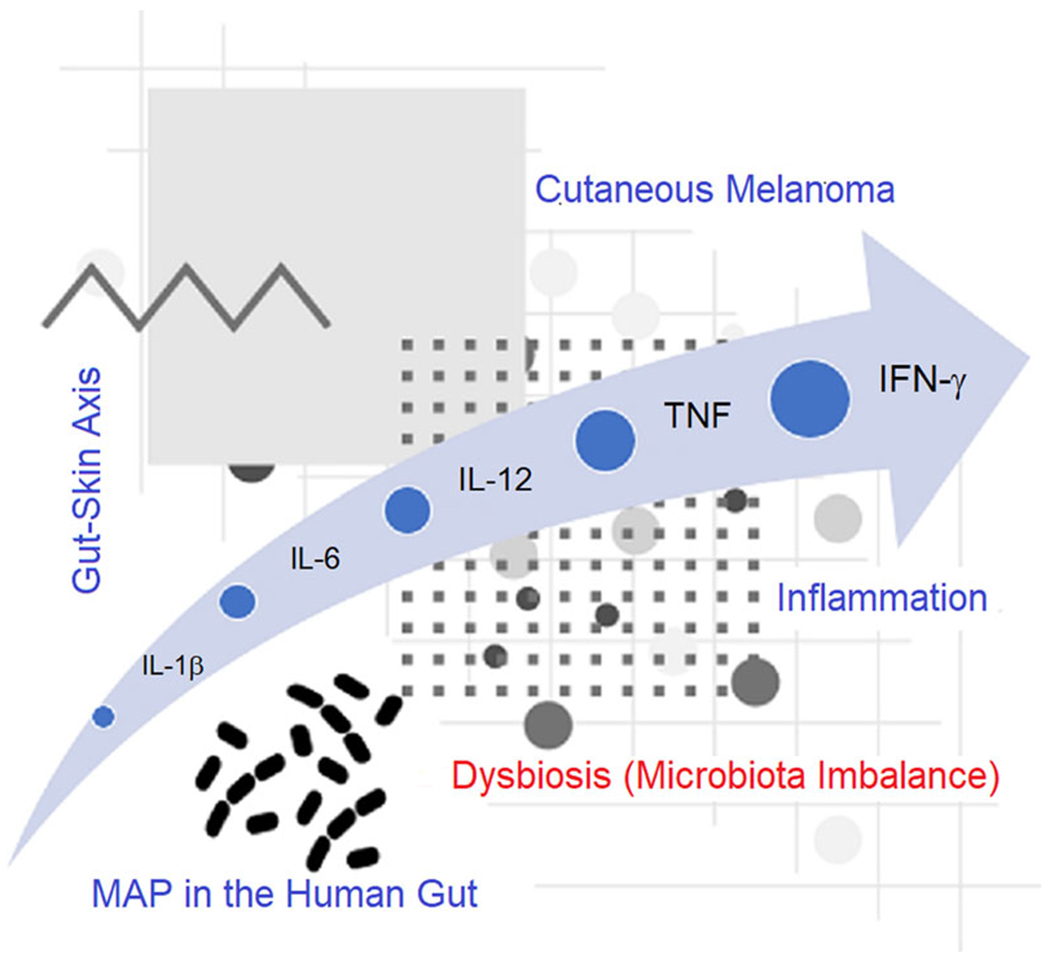
Importance of the gut–skin axis in the development of cutaneous melanoma. Patients with high levels of inflammatory cytokines, for example, interleukin-1β (IL-1β), interleukin-6 (IL-6), interleukin-12 (IL-12), tumor necrosis factor (TNF), and interferon-*γ* (IFN-*γ*) may result from the “challenging to clear” *Mycobacterium avium* subspecies *paratuberculosis* (MAP) in the gut. This manifests as decreased integrity of the gut epithelium and microbiota imbalance (dysbiosis). Chronic inflammation, disruption of the gut–skin axis, and dysbiosis are important etiologic determinants of cutaneous melanoma risk.

MAP is believed to readily colonize the gut, as illustrated by its appearance in patients with Crohn’s disease, and to a lesser degree in those without this condition.^184^ This bacterium is notoriously persistent and difficult to clear with anti-MAP antibiotics, often taking many months or years when effective to eradicate the organism from the gut and body. Reported infection and prevalence rates of this nonspore-forming microbe likely are underestimated given the paucity of routine testing and standard testing methodology. Environmental sources for gut MAP are ubiquitous, including the consumption of infected dairy foods, underdone meat, and drinking or encountering infected soil and water. In many cases, the organism is resistant to pasteurization, further exasperating public health control efforts. Various inflammatory skin conditions such as psoriasis, atopic dermatitis, and rosacea have been associated with bacteria in the gut, with skin cancer also hypothesized to belong to this list of disorders.^185^ Patients with serum bacterial DNA presumed to originate from the intestinal lumen specifically manifest high levels of inflammatory factors (e.g., IL-1β, IL-6, IL-12, tumor necrosis factor, and interferon *γ*), suggesting a decreased integrity of the gut epithelium and overall negative homeostasis (Figure 4).^186,187^

The “gut-skin axis,” vis-á-vis dysbiosis (microbiota imbalance), has been postulated to underlie CM by impairing the intestinal barrier. This is characterized by systemic inflammation, oxidative stress, and maladaptive redox processes, potentially facilitating tumor progression and acquired chemoresistance of skin cancer.^188^ Following ingestion, MAP appears to penetrate the intestinal wall by way of the ileal mucosa but also can cross the gut mucosa through enterocytes, independently of Peyer’s patches.^189^ This Gram-positive, acid-fast bacterium is resistant to intracellular degradation. Infected macrophages easily spread to the lymphatic system to impair immunologic responses and manifest as increasing amounts of adhesion molecules and the formation of granulomas (e.g., KdpFABC complex).^189^ MAP can obstruct superoxide anions, hydrogen peroxide, and hydroxyl radicals to affect immune modulation (interleukin-10/interleukin-12, and interleukin-23). The virulence of MAP is intrinsically related to its ability to acquire nutrients within the gut and break down host fatty acids. The long-term survival of MAP within host macrophages is associated with genetic modifications linked to iron limitation and nitric oxide build-up.

Imbalances in gut microbiota have been correlated with incipient melanoma and weakened response to immune therapies.^190^ Bacterial profiles enable the risk stratification and discrimination of melanoma from non-melanoma, with differences observed in the structure and richness of colonies in patients with early-stage melanoma. Although MAP is not specifically mentioned, similar gut bacteria appears to affect immune surveillance and cancer development. By analogy, one may infer that gut MAP increases the level of circulating pro-inflammatory cytokines and in effect prompt immune cells to migrate toward the skin, resulting in local inflammation, a key step in the neoplastic development of melanocytes.

While the gut represents the primary home of human commensal microbiota, microbial toxins operating within the skin (the largest organ of the body) may facilitate chronic inflammation and cell damage, precipitating melanoma development and progression.^185^ As the skin comes in direct contact with sunlight, it has been further suggested that the deleterious effect of UV radiation-induced immunosuppression is intensified in the presence of certain cutaneous bacteria. In theory, UV-radiation (a pivotal carcinogen) may interact with MAP to compromise the microenvironment of CM, with important disruptions to CD (cluster of differentiation)8+ T-cells, regulatory T-cells, cytokines, chemokines, and tumor-associated macrophages. If not countered by the presence of “good” bacteria to promote intestinal health and positive vitamin D levels, local UV-induced changes to skin microbiota may exasperate “bad” gut microbiota and vice versa, through systemic pathways.^186^

Several immunotherapeutic advances, such as anti-programmed cell death 1 (anti-PD-1), anti-programmed death-ligand-1 (anti-PD-L1), and anti-cytotoxic T-lymphocyte-associated antigen 4 (anti-CTLA-4) have become available to better manage moderate to advanced-staged melanoma.^191,192^ However, the positive response to these compounds is limited to a subsegment of the melanoma population and even then may not be durable over time. One explanation posits that innate and adaptive immunity may be influenced by the gut microbiome, wherein variations in bacteria colonies differentially effect treatment outcomes and frequency of immune-related adverse effects. That is, responders (Rs) versus non-responders (NRs) may manifest differences in terms of their gut microbiome balance. Research efforts aiming to optimally adjust the gut microbiota to improve the therapeutic benefit of these immunologic agents include fecal microbiota transplantation, probiotics, and diet. While such attempts mainly have focused on enriching the microbiota with so-called “good” bacteria, there has been a dearth of research aimed at measures to directly minimize the negative load of harmful bacteria in the gut. One possible avenue for future study would be to assess the potential benefit of RedHill Biopharma (RHB)-104, an anti-MAP mix of antibiotics. While such an approach likely would spill over to eradicate beneficial bacteria colonies, the post-administration of probiotics might be required to replenish healthful bacteria.

### 2.7 | Genetic factors

**FIGURE 5:**
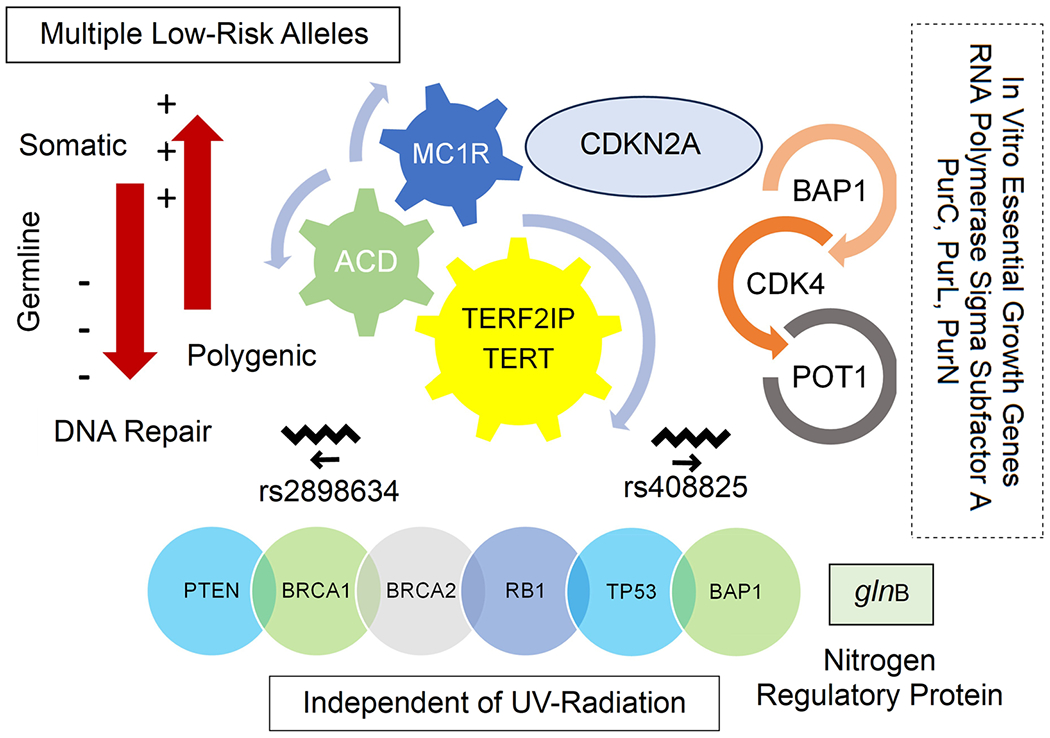
The role of genetics in cutaneous melanoma development. Genetics have an indirect role in melanoma susceptibility, suggesting an interaction with environmental exposures (e.g., *Mycobacterium avium* subspecies *paratuberculosis* [MAP]). Many genes are independent of ultraviolet (UV) radiation such as phosphatase and tensin homolog (PTEN), breast cancer type 1 susceptibility protein (BRCA1), breast cancer type 2 susceptibility protein (BRCA2), retinoblastoma protein 1 (RB1), transformation-related protein 53 (TP53), BRCA1 associated protein 1 (BAP1), and glutamine synthetase gene-B (*gln*B). Typically, most variants associated with melanoma are somatic; however, a polygenic mode of inheritance involving the interplay of multiple low-risk alleles or rare mutations in high-penetrance genes such as adrenocortical dysplasia protein homolog (ACD), BAP1, cyclin-dependent kinase 4 (CDK4), cyclin-dependent kinase inhibitor 2A (CDKN2A/p16INK4a), protection of telomeres 1 protein (POT1), telomeric repeat binding factor 2 interacting protein (TERF2IP), and telomerase reverse transcriptase (TERT) should not be ruled out. In non-pigment pathways, DNA repair, as suggested for melanocortin 1 receptors (MC1R), may underlie melanoma predisposition. Additionally, in vitro growth genes important to melanoma development include ribonucleic acid (RNA) polymerase sigma subfactor A (sigA), phosphoribosylaminoimidazole-succinocarboxamide synthase (PurC), phosphoribosylformylglycinamidine synthase subunit (PurL), and phosphoribosylglycinamide formyltransferase 1 (PurN). The significant genome-wide association with rs28986343 at the human leukocyte antigen (HLA) locus and the association between rs408825 and expression of the innate immunity gene interferon-induced guanosine triphosphate (GTP)-binding protein (MX2) supports an immunologic role for melanoma development.

Approximately 90% of individuals presenting with CM do not have any affected relatives, suggesting that inheritance only plays a minor role in the direct development of melanoma.^9^ Rather, in most cases, genetics appear to have an indirect role, interacting with environmental factors (possibly MAP) to increase melanoma susceptibility through inflammatory and various other growth pathways. While most genetic variants associated with melanoma risk are somatic in nature, this does not rule out a polygenic mode of inheritance involving the interplay of multiple low-risk alleles or rare mutations in high-penetrance genes (e.g., ACD, BAP1, CDK4, CDKN2A, POT1, TERF2IP, TERT). A link with DNA repair, as suggested for melanocortin 1 receptors (MC1R), may underlie melanoma predisposition, especially in non-pigment pathways (Figure 5).

A recent meta-analysis of 36,760 melanoma cases identified an increased risk for 68 independent SNPs, corresponding to 54 loci.^193^ Although many of these SNPs were associated with “pigmentation, tanning response, nevus count, and telomere maintenance”, a subset of sites diagnosed in populations at low risk for UV-induced melanomas (e.g., nail beds, palms, soles), appear to be unrelated to pigmentation. Furthermore, in an extended pleiotropic analysis, many loci were not associated with nevus count or hair color, suggesting that genetic variants often “act outside of … classic cutaneous melanoma risk phenotypes.” Additionally noted, an immunologic role for melanoma risk was supported by the significant genome-wide association with rs28986343 at the HLA locus, in line with previous studies indicating an “association between rs408825 and expression of the innate immunity gene *MX2*.” Interestingly, several risk alleles for vitiligo, an autoimmune melanocyte-related disorder, are protective for CM, highlighting the important role of immunity in the development of CM.

The evasion of immune response has been associated with BRAF mutations “affecting signaling pathways in melanoma development.”^194^ Abnormal BRAF kinase activity occurs in approximately half of CMs owing to point mutations at codon 600, with the replacement of glutamic acid for valine. BRAF mutations often arise in tumors that are free of “chronic sun induced damage.” While this raises the possibility of a nonsolar role for MAP in the etiology of melanoma, there are no studies to date that support this supposition. Overall, the immunology of melanoma is complex with many redundant pathways. Targeted immune-based therapy has shown success in a subset of melanoma skin cancer patients. However, acquired resistance is a commonly observed outcome.

Independent of UV radiation, CMs often present in mixed cancer syndromes attributable to “mutations in PTEN, BRCA2, BRCA1, RB1, and TP53.”^195^ The “epithelioid cytology of melanocytic tumors may suggest an underlying BAP1 mutation,” frequently characterized as BAP1 tumor predisposition syndrome (BAP1-TPDS). This syndrome is associated with several tumors of nonsolar origin, such as renal cell carcinoma, mesothelioma, and posteriorly occurring uveal melanomas. The nonsolar role of MAP in posteriorly located uveal melanoma has been recently explored in a narrative review on this topic.^34^

The genetic basis for the survival and persistence of MAP in a host organism is an important requisite for skin cancer development. A recent “Hidden Markov Model” analysis identified 430 in vivo genes essential for colonization in the natural host and 260 in vivo growth-defect genes.^196^ Furthermore, a gene ontology (GO) enrichment analysis “revealed an overrepresentation of genes for biological process, molecular function, and chemical component categories,” with top-ranked GO terms suggesting the involvement of “in vivo essential genes in DNA repair, pathogenesis, symbiosis and parasitism growth, response to stimuli and oxidation-reduction processes.” In particular, the transcription initiation factor “RNA polymerase sigma subfactor A (sigA)” was identified an in vitro essential gene, while genes deemed to underlie in vitro growth of MAP and H37R*v* included “PurC, PurL, and PurN,” vis-á-vis biosynthesis of arginine, aromatic amino acids, ATP, folic acid, purine, and riboflavin.

While the above critique has largely focused on chronic intestinal inflammation attributable to MAP in ruminants, one may hypothesize that a similar set of genes underlie colonization, persistence, and survival of mutant MAP in the gut and skin of human hosts. For example, the upregulation of SigA has been noted as an in vivo growth promoter in the “sister” organism MTB in humans. Similarly, the open reading frame (ORF) R*v*031 for human MTB was mentioned as a virulent promoter of “bacilli survival in harsh conditions and inside macrophages.”^197^ Another gene *glnB*, believed to encode a nitrogen regulatory protein, also has been purported to resist “the IFN-*γ*-activated macrophage” environment in human MTB,^198^ curtailing a key host defense of MTB as a persistent infection. As well, mutations among individuals in the Toll-like receptors (TLRs) manifest increased susceptibility to mycobacteria, wherein TLR9 specifically is believed to “initiate responses that are critical in defense against MAP.”^199^

### 2.8 | Other means of evading the immune system

As evolutionary genetic processes have allowed MAP to efficiently infect host animals and humans, a cascade of nongenetic mechanisms conceivably are involved as well to evade immune response and dysregulation of cellular metabolism. Of importance, MAP “in human cells are cholesterol dependent” wherein “these bacteria localize to cholesterol-rich compartments that are slow to acidify.”^200^ The persistence of MAP allegedly has been linked to interruption of phagosome acidification and the “ineffective recruitment of RAS-associated binding7 (Rab7)-interacting lysosomal protein (RILP) to the phagosomal membrane.” Furthermore, MTB Mce3e has been reported to suppress innate immune responses through the targeting of ERK1/2 signaling and MTB Rv2387 appears to enable bacterial survival through TLR2/p38/JNK signaling.^201^

### 2.9 | Vaccine relationship

**FIGURE 6:**
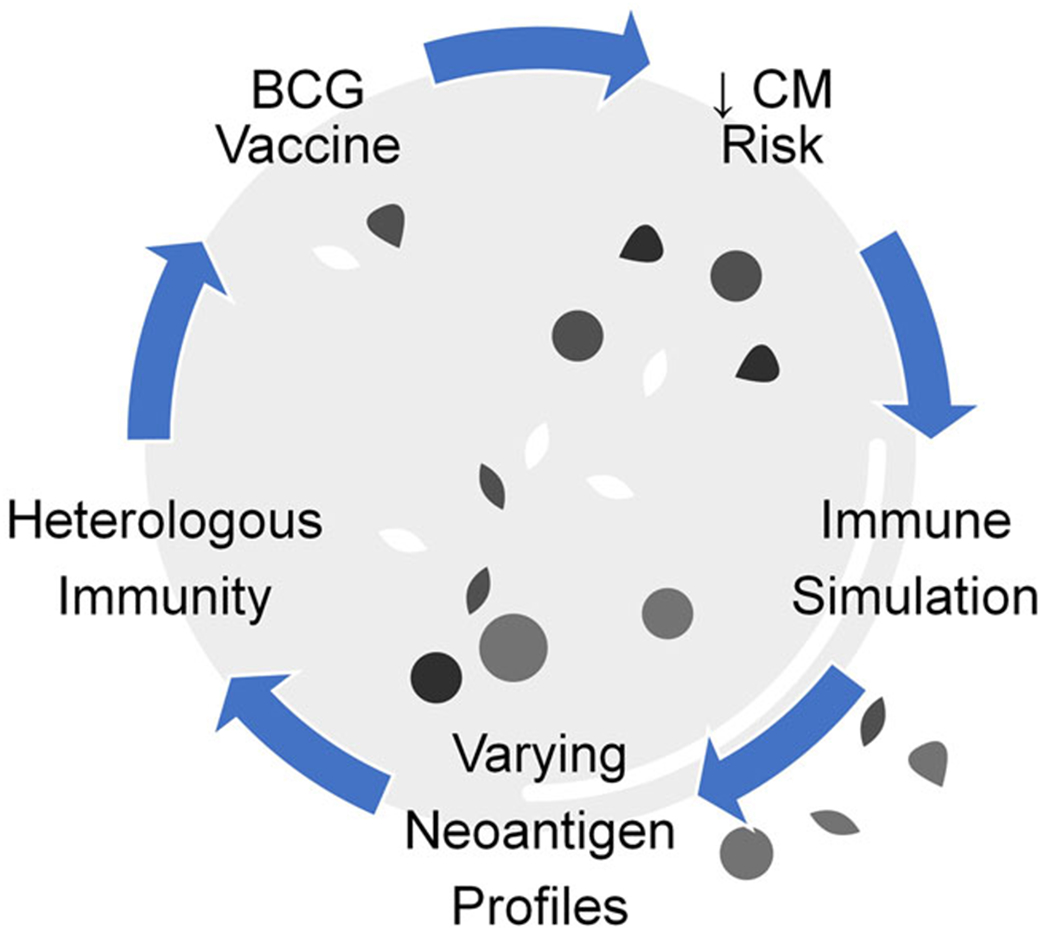
Immune system role in cutaneous melanoma risk and therapy. BCG vaccination potentially may act through the simulation of heterologous or trained immunity against *Mycobacterium avium* subspecies *paratuberculosis* (MAP). However, antigens vary among melanoma patients, presenting an obstacle to the development of individualized immune-related therapies.

The BCG vaccine for tuberculosis has been proposed and occasionally documented to effectively treat MAP-associated autoimmune diseases.^202^ BCG may have similar effects against CM. Vaccinations in general and the BCG vaccination in particular confer a lower risk for CM.^203^ In the case of vaccinations other than BCG, vaccination may act through the stimulation of heterologous or trained immunity against unrelated organisms.^202^ The BCG vaccine in contrast may act by the stimulation of immunity against another species of bovine mycobacteria. As previously mentioned, the BCG vaccine is an attenuated *Mycobacterium bovis* virus. In addition to lowering the risk of CM, the BCG vaccine also has been used to successfully treat CM by being injected into the lesions (Figure 6).^204–206^

The successful treatment of CM with intralesional BCG further supports an association between MAP and CM. BCG is widely used throughout Asia as part of childhood immunization programs^207^ and may underlie the low rates of CM in this population.^208^ Although the BCG vaccine is not routinely administered in European countries such as the United Kingdom, it is occasionally used prophylactically to offset tuberculosis in high-risk individuals. However, it is not known if the rate of CM is lower among Britons who were vaccinated.

## 3 | ANIMAL MODELS

Animal models of melanoma using nude mice have focused almost exclusively on the possible role of UV radiation in melanoma development.^209,210^ We propose instead that dogs rather than mice will be excellent models of MAP-associated human CMs for the following reasons.

As discussed above, in countries where MAP infection of domestic livestock is longstanding, MAP is present throughout the soil of the country, in areas of both high and low density of cattle.^93^ The presence of MAP in soil may explain the development of CM in dogs, who may be infected with MAP from their near field exposure to MAP-contaminated soil. Most dog species bodies are covered by hair presumably minimizing the risk of melanoma related to UV exposure. Conversely, this coverage creates more opportunities for MAP to persist on canine hair and skin.

Since both human and canine CM may be caused by MAP, canine CM may be an excellent model for study of human CM not attributed to UV radiation.^211–213^ One review of animal models of human cancers highlights canine and humans’ common environmental exposure to a “broad array of pathogens.”^214^

## 4 | LIMITATIONS AND STRENGTHS OF THE EVIDENCE

A recent review of the more well-known association of MAP with Crohn’s disease discusses the many limitations in establishing MAP as the cause of human disease.^25^ Detecting the organism in human tissue is especially difficult owing to the variability in MAP detection methods and the putative existence of MAP in a cell wall deficient form that is not amenable to the usual mycobacterial stains. Systematic reviews of the detection of MAP in patients with Crohn’s disease have generally supported a causative association.^24,215,216^

MAP has been identified in human blood, intestinal tissue, and lymph nodes, but not in human skin. Porcine skin^217,218^ and human melanocyte cell lines^219^ are available to test MAP’s ability to penetrate skin and melanocytes, and the effect of MAP on these tissues and cells. Two studies have documented the presence of MAP on the surface of beef cattle hides but not within the epidermis.^220,221^

While intriguing, the hypothesis that MAP exposures and CM are associated may not formally meet all nine criteria of Hill’s correlates for causation (strength of association, consistency, specificity, temporality, biological gradient, plausibility, coherence, experiment, and analogy). However, these correlates are only guidelines. For example, A may cause B, yet may not necessarily meet all of Hill’s criteria. Alternatively, a risk factor that meets all criteria may be determined to be a confounder rather than a true cause.^222,223^

There is no research available that studies CM and immunosuppression involving MAP. Ideally this review will stimulate such research. As well, CM manifests significant intratumor and intertumoral heterogeneity, and varies by pathologic features (e.g., Breslow depth), transcriptomics, proteomics, and genomics.^224^ In the latter case, there are at least four genetic subtypes of melanoma (e.g., mutant BRAF, mutant RAS virus, mutant neurofibromatosis type 1 [NF1], and Triple-Wild-type) which may differentially interact with MAP.^225^

While it may be argued that the current body of scientific literature does not provide adequate evidence of a direct link between MAP and melanoma, one is reminded of the now well-accepted causal association between *Helicobacter pylori (H. pylori)* and gastric cancer, which was similarly criticized for years as lacking consistent evidence. The current manuscript is the first time a possible causative association has been proposed between MAP and CM, and thus there are no epidemiologic studies or systematic reviews of this association. The more well known possible etiologic association between MAP and Crohn’s disease has, in contrast, been the subject of numerous epidemiologic studies and systematic reviews. Despite the consistent evidence from these studies and reviews, MAP is still not generally accepted as a cause of Crohn’s disease, highlighting the similar difficulty of establishing a link between MAP exposure and CM.

The relationship of CM with sun exposure is complicated and only partially explained by current scientific reasoning (e.g., intermittent exposure and dual pathway models).^226^ Arguments contrary to a definitive cause and effect association for UV-radiation are multifold. This includes (1) a relatively small increase in incidence with age compared with squamous and basal cell skin cancers, (2) the historical predominance in women versus men, (3) an incongruence with areas of the body more versus less exposed to sunlight, (4) the comparatively rare occurrence of CM among black albinos, and (5) only marginally increased incidence with latitudes more proximal to the equator versus squamous and basal cell skin cancers.^227^ Accordingly, other nonsolar risks for CMs, such as MAP, are worth considering.

Exposure to MAP does not mean that one will develop melanoma, but rather that certain susceptible individuals may have an increased risk of developing the cancer, possibly years after the bacterium has been successfully eradicated from the body. Such is the case for *H. pylori* as a cause of gastric cancer, which has been demonstrated as long as 14 years after elimination of the bacterium.^228^ However, reinfection is a constant concern for both *H. Pylori* and MAP, requiring routine surveillance to assure the effectiveness of initial antibiotic therapy. Even then, primary and tertiary measures may only halve the cancer risk.^229^ Like *H. Pylori* infection and gastric cancer, only a fraction of MAP-exposed individuals potentially will manifest melanoma.

Since the hypothesis that MAP may cause some cases of human CM is new, there have been no efforts to identify MAP in human CM lesions. Of interest, Nobel laureate Zur Hausen discusses how the injection of the vaccinia virus into human skin has been associated with the subsequent development of CM at the injection site, and speculates that a “cattle virus” contaminating the vaccinia vaccine may be responsible for the CM.^58,230^ *Mycobacterium leprae* (ML) organisms have been identified in a basal cell carcinoma and a CM in a patient with ML, probably attributable to subsequent contamination of the already existing cancers with the ML organism.^231^ In the context of a previous discussion of the possible relationship between MAP and uveal melanoma, the ability of MAP to invade various human cell types, including enterocytes, macrophages, dendritic cells, and small intestinal goblet cells, was noted.^34^

While genomic epidemiologic methods have advanced considerably in recent years for detecting MAP in isolates of ruminants, human detection still remains a challenge.^232^ The case in point being the lack of a gold standard such as culture upon which to validate results, appropriate guidance on best practices for selecting and handling human tissue specimens, and methods for the identification of referent groups by geography and prevalence of MAP in the environment are needed. The availability of targeted polymerase chain reaction (PCR) probes, or their mismatch, remains another concern given the genetic similarity of MAP with other mycobacteria of the *Mycobacterium avium* family.^233^

Establishing causality for a bacteria and cancer is difficult, especially when the determination of exposure is made at the point of diagnosis.^234^ Retrospective analyses are prone to the misclassification of cases as bacteria negative when individuals were infected in the past and the infection has since cleared. In lieu of costly randomized cohorts, nested case-referent studies present an alternative means of exploring the association between MAP and CM, although this design is subject to specimen degradation and differential attrition of the cohort over time.

## 5 | CONCLUSIONS

Melanoma skin cancer typically has been posited to result from exposure to sunlight as a source of UV radiation. This belief has limited the exploration of other hypothesized causes, especially where melanoma appears on the palms of the hands, soles of the feet, and on other areas of the body less exposed to the sun. The occurrence of primary gastric melanoma also raises questions about the exclusive role of sunlight in causing melanoma. These questions illustrate the need for further discussions on other possible causes of melanoma such as MAP.

When considered together, the evidence for MAP as a causative factor is not yet conclusive, with plausible alternative explanations. Future efforts will need to move beyond case examples that simply provide ecologic and patient characteristics at a single point in time. More epidemiologically rigorous and well-powered, population-based study designs are essential, as well as identifying best practices for the prevention and treatment of bacterial infections such as MAP. Lastly, individuals with MAP exposure may be susceptible to many different microbes having carcinogenic potential. Disentangling such etiologic agents will be key to determining if MAP is an independent risk factor for CM versus a coincidental risk.

In summary, the MAP hypothesis provides a new perspective on the pathogenic mechanisms underlying CM leading to better awareness of this disease. Ideally, the consideration of MAP as an etiologic agent may yield more effective prevention strategies and targeted treatment alternatives for CM.

## Data Availability

Not applicable.
